# How to test for partially predictable chaos

**DOI:** 10.1038/s41598-017-01083-x

**Published:** 2017-04-24

**Authors:** Hendrik Wernecke, Bulcsú Sándor, Claudius Gros

**Affiliations:** Institute for Theoretical Physics, Goethe University Frankfurt, Frankfurt am Main, Germany

## Abstract

For a chaotic system pairs of initially close-by trajectories become eventually fully uncorrelated on the attracting set. This process of decorrelation can split into an initial exponential decrease and a subsequent diffusive process on the chaotic attractor causing the final loss of predictability. Both processes can be either of the same or of very different time scales. In the latter case the two trajectories linger within a finite but small distance (with respect to the overall extent of the attractor) for exceedingly long times and remain partially predictable. Standard tests for chaos widely use inter-orbital correlations as an indicator. However, testing partially predictable chaos yields mostly ambiguous results, as this type of chaos is characterized by attractors of fractally broadened braids. For a resolution we introduce a novel 0–1 indicator for chaos based on the cross-distance scaling of pairs of initially close trajectories. This test robustly discriminates chaos, including partially predictable chaos, from laminar flow. Additionally using the finite time cross-correlation of pairs of initially close trajectories, we are able to identify laminar flow as well as strong and partially predictable chaos in a 0–1 manner solely from the properties of pairs of trajectories.

## Introduction

One characteristic aspect of deterministic chaos is the exponential sensitivity of the dynamics to initial conditions^1, 2^. This sensitivity leads to an effective breakdown of predictability as the result of the eventual decorrelation of any given pair of trajectories. The decorrelation occurring on a chaotic attracting set is measured commonly by the maximal Lyapunov exponent^3^, with a positive value measuring the effective rate of the decorrelation of initially arbitrary close pairs of trajectories. Other standard tests for chaos, such as the correlation dimension^4^ or the spectral analysis of the auto-correlation function^5^, also rely on correlation measures.

Inter-orbital correlations fully decay on chaotic attractors in the limit of long times. This fact does however not preclude the existence of other types of predictable correlations. E. g. it is well known^6^ that the sequence *x*_*n*_ produced by the logistic map *x*_*n*+1_ = *rx*_*n*_(1 − *x*_*n*_) will never decrease twice in a row for 2 < *r* < 4. In this case we can hence predict with 100% confidence that *x*_*n*+1_ will be larger than *x*_*n*_, if *x*_*n*_ was smaller than *x*_*n*−1_, even if the system is chaotic. It has also been noted that correlations may persist for specific chaotic systems for extended (but finite) times, especially in systems characterized by multiple time scales^7^ or strong periodic drivings^8^. The respective cross-correlation *C*_12_ of initially close pairs of trajectories can hence be used as a measure for predictability.

In the following we will introduce, distinguish and discuss two types of chaotic behavior, denoted partially predictable chaos (PPC) and strong chaos respectively, which differ with respect to what happens for time scales larger than the Lyapunov prediction time *T*_*λ*_.

- *Strong chaos*: Predictability vanishes, approaching zero on a time scale of *T*_*λ*_.
- *PPC*: The first decorrelation occurring on a time scale of *T*_*λ*_ does not destroy, in this case, all pair-wise correlations. The cross-correlation *C*_12_ will retain a finite value even for *t* ≫ *T*_*λ*_, vanishing only for exceedingly long times.

Partial predictability may occur whenever the attracting set is characterized by a non-trivial topology. This is generically the case for the chaotic state close to a period-doubling transition, when the trajectories wander chaotically around previously stable limit cycles within closed braids^1^. Our observation of partial predictability may hence be especially of relevance for natural systems having a tendency to self-organize close to criticality^9^, as it implies that the system will hover continuously close to the transition between laminar and chaotic flow^6^. Another example of PPC is the case of phase locked chaos observed in driven Josephson junctions^10, 11^.

It is generically a challenge to distinguish between partially predictable chaos and laminar flow on the basis of the maximal Lyapunov exponent, which is positive but small for PPC and hence difficult to evaluate numerically. We therefore introduce here a novel test for chaos based on the cross-distance scaling of pairs of trajectories. It discriminates chaos, including PPC and strong chaos, unambiguously in a 0–1 manner. In the following we provide a theoretical motivation for the test, a comparison with other measures and an application to the Lorenz system^12^, for which we find all three types of dynamical regimes: strong chaos, PPC and laminar flow.

Thereafter we combine the indicator for chaos with another correlation indicator acting as an effective 0–1 test, namely the finite time cross-correlation of initially close trajectories, that is able to distinguish strong chaos from PPC. The combination of both indicators is capable of drawing an unambiguous distinction between all three dynamical phases. As both tests are based on pairs of initially close trajectories, requesting only straightforward data manipulation, they are easy to implement and suitable both for a wide range of scientific fields and for a possible automation of the procedure.

## Results

We start by considering with **x** = (*x*, *y*, *z*),

1 $$\dot{x}=\sigma (y-x),\quad \quad \dot{y}=x(\rho -z)-y,\quad \quad \dot{z}=xy-\beta z$$

the Lorenz system^12^, which has long been used for studying the interplay between predictability and chaos^13–15^. We select with *β* = 8/3 and *σ* = 10 standard parameter settings, retaining *ρ* as the bifurcation parameter.

**Figure 1:**
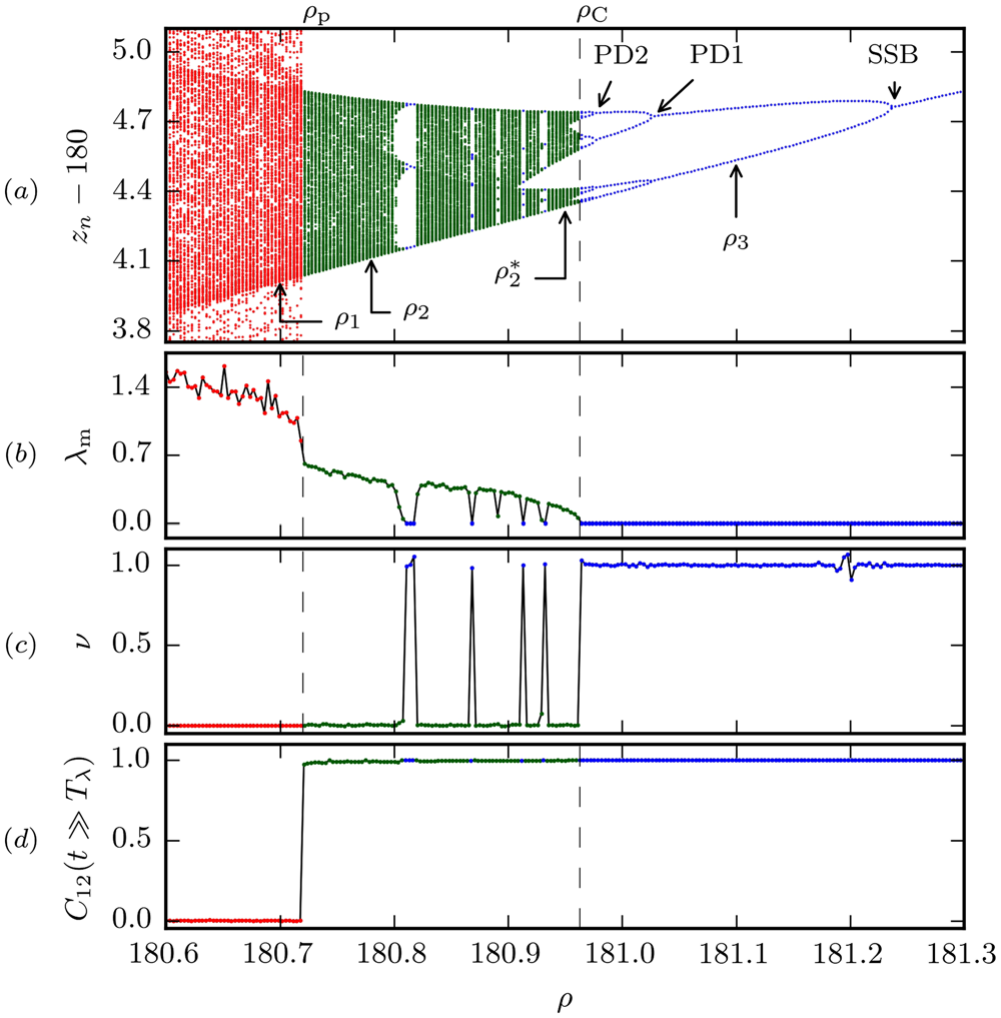
Strong chaos (red, for *ρ* < *ρ*_p_ ≈ 180.72), partially predictable chaos (green, for *ρ*_p_ < *ρ* < *ρ*_C_ ≈ 180.96) and regular flow (blue, for *ρ*_C_ < *ρ*), for the Lorenz system (1). Shown are (from top to bottom), *z*_*n*_ from the Poincaré section *x* = 15 (for a window of *z*_*n*_), the maximal Lyapunov exponent *λ*_m_, the cross-distance scaling exponent *ν*, see Eq. (2), and the cross-correlation *C*_12_(*t* = 200), as defined by Eq. (3). PD1 and PD2 denote examples of period doubling bifurcations and SSB a bifurcation spontaneously breaking the symmetry (*x*, *y*, *z*) ↔ (−*x*, −*y*, *z*) of (1).

As an overview we present in Fig. 1 the phases of the Lorenz system for a typical parameter window *ρ* ∈ [180.6, 181.3]. A transition between two types of chaotic regions is observed to occur at *ρ*_p_ ≈ 180.72, together with a transition via a cascade of period doubling (halving) bifurcations from chaos to laminar flow at *ρ*_C_ ≈ 180.96. The dynamics of the intermediate region (*ρ* ∈ [*ρ*_p_, *ρ*_C_], green) between strong chaos (*ρ* < *ρ*_p_, red) and laminar flow (*ρ* > *ρ*_C_, blue), is governed by PPC. A spontaneous symmetry-breaking bifurcation^16^ (SSB) is additionally shown. Chaos-chaos transitions involving phase space explosions, like the one occurring at *ρ*_p_ between partially predictable and strong chaos (which is in part intermittent^17^), have been studied previously in the context of quadratic maps^18^ and driven Josephson junctions^10, 11^. They are due to the collision of an unstable manifold with the attracting chaotic set, an interior crisis^18^ which is a typical example of a global bifurcation^6^.

**Figure 2:**
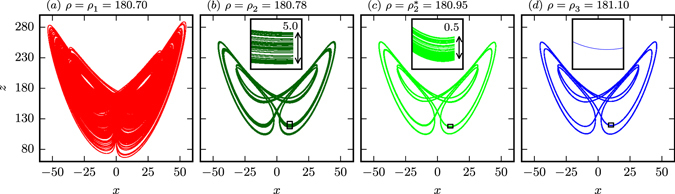
Sample trajectories of the Lorenz system (1) projected to the *x* − *z* plane. The phases, from left to right, *ρ* = *ρ*_1_ (ergodic motion), *ρ* = *ρ*_2_ and $\rho ={\rho }_{2}^{\ast }$ (partially predictable chaos) and *ρ* = *ρ*_3_ (limit cycle), are indicated in Fig. 1. The arrows in the respective insets indicate the width of the (fractal) braids in the partially predictable phase. The respective standard deviations *s* of the attractors are *s* ∈ [58.76, 58.92] for *ρ* ∈ [*ρ*_1_, *ρ*_3_].

In Fig. 2 we present the projections to the *x* − *z* plane of the respective attracting sets for (*a*) strong chaos, (*b*) and (*c*) PPC, and (*d*) laminar flow. Partially predictable chaos is at times difficult to distinguish visually from laminar flow (compare Fig. 2(c,d)), we hence provide the respective blow-ups in the insets. The partially predictable chaotic attractors can be thought as fractally broadened limit cycles, viz as braids.

The maximal Lyapunov exponent^19^ *λ*_m_ presented in Fig. 1(b) has been evaluated by extracting the initial slope of the logarithmic distance 〈ln|**x**_1_(*t*) − **x**_2_(*t*)|〉 of two trajectories, as averaged over 10^4^ pairs with initial distances of *δ* = 10^−8^, when plotted as a function of time. |…| denotes here the Euclidean distance and 〈…〉 the average over initial conditions on the attractor sampled with the natural distribution^1^ (the natural invariant measure^20^). We used in addition 10^4^ pairs of trajectories for the cross-correlation *C*_12_(*t* = 200), see Eq. (3). The choice of *t* = 200 has been made in order to ensure that we neither have to deal with initial effects nor with numerical inaccuracies, the latter due to the chaotic nature of the flow.

We have also evaluated a scaling exponent *ν* (discussed further below, see Eq. (2)), which characterizes the scaling of the long-term distance between two trajectories. Our choice to favor the average logarithmic distance for computing the maximal Lyapunov exponent over more sophisticated methods is motivated by a conceptual computational aspect: in this way all three indicators presented here, i. e. the maximal Lyapunov exponent *λ*_m_, the cross-correlation *C*_12_ and the cross-distance scaling exponent *ν*, can be evaluated from the time evolution of initially close-by trajectories. In the Methods section we compare this approach for computing the maximal Lyapunov exponent to the results obtained by Benettin’s method^21, 22^.

Two fundamental time scales determine the initial dynamics. The first is the quasi-period *τ*, which is the average time a trajectory needs to come back to the same intersection of the braid with the Poincaré plane. It is comparable to the period of the limit cycle and we find *τ* ≃ 2.2 to hold for all partially predictable attractors *ρ*_C_ < *ρ* < *ρ*_p_. The second time scale is the Lyapunov prediction^23^ time *T*_*λ*_ = ln(|**x**_1_ − **x**_2_|/*δ*)/*λ*_m_, which is the time it takes for two exponentially diverging trajectories starting from an initial separation *δ* to reach a given finite distance |**x**_1_ − **x**_2_|. For these two distances we used *δ* = 10^−8^ and |***x***_1_ − **x**_2_| ~ 0.001 respectively. At the latter distance a finite amount of predictability is lost, viz the cross-correlation *C*_12_, as defined by Eq. (3), starts to deviate from unity. Given the values of the maximal Lyapunov exponent *λ*_m_ presented in Fig. 1 we obtain *T*_*λ*_ ≈ 10 and *T*_*λ*_ ≈ 25 for *ρ* = *ρ*_1_ = 180.70 and *ρ* = *ρ*_2_ = 180.78 respectively (cf. also Table 1). The initial loss of predictability occurs hence after a few cycles around the braid.

**Table 1:** The average maximal Lyapunov exponent *λ*_m_ together with the second- and third largest Lyapunov exponent, *λ*_2_ and *λ*_3_ (cf. Methods section).

	*λ*_m_	*λ*_2_	*λ*_3_	*T*_*λ*_(*δ*)	
				*δ* = 10^−5^	*δ* = 10^−8^
*ρ*_1_ = 180.70	1.18	0.00	−14.85	3.9	9.8
*ρ*_2_ = 180.78	0.47	0.00	−14.14	9.8	24.5
${\rho \,}_{2}^{\ast }=180.95$	0.14	0.00	−13.81	32.9	82.2
*ρ*_3_ = 181.10	0.00	−0.60	−13.07	—	—

**Figure 3:**
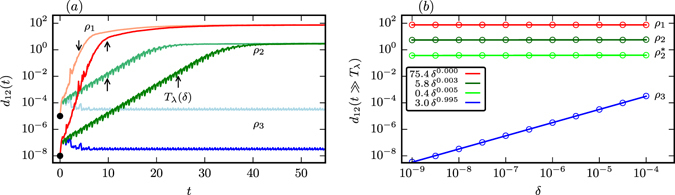
Distance scaling of initially close-by trajectories. (**a**) The distance *d*_12_(*t*) of initially close-by pairs of trajectories, averaged over 10^4^ initial conditions, with initial distances *d*_12_(0) = *δ* = 10^−8^ and *δ* = 10^−5^ (black bullets). For the regular flows (*ρ* = *ρ*_3_ = 181, blue lines) the long-term distance depends on *δ*. The strongly chaotic attractor (*ρ* = *ρ*_1_ = 180.7, red lines) approaches the maximal distance independently of the initial distance within the respective Lyapunov prediction time *T*_*λ*_. In the case of PPC (*ρ* = *ρ*_2_ = 180.78, green lines) the distances reach a quasi stationary plateau that is independent of the initial distance. For comparison we marked the Lyapunov prediction times *T*_*λ*_ at the respective curves by arrows. (**b**) The scaling behavior, see Eq. (2), of the averaged long-term distance *d*_12_(*t* = 200). The results (circles) are for the strongly chaotic phase (*ρ* = *ρ*_1_, top), for the partially predictable chaos (*ρ* = *ρ*_2_ and $\rho ={\rho \,}_{2}^{\ast }$, middle) and for a limit cycle (*ρ* = *ρ*_3_ = 181.10, bottom), as indicated in Fig. 1. The respective solid lines are linear fits to the log-log plot.

The respective Lyapunov prediction times *T*_*λ*_ have been evaluated for the two sets of initial distances *δ* used in Fig. 3. The values of *ρ* used are indicated in Fig. 1.

### Cross-distance scaling

A large body of work^3, 4^ has shown that strange attractors are relatively difficult to characterize in detail, even for low-dimensional dynamical systems. For systems with a higher dimension, such as autonomous neural networks or climate models, it may even be a challenge to robustly distinguish laminar from chaotic flows. Here we propose that the scaling of the long-term distance *d*_12_(*t* ≫ *T*_*λ*_) of two trajectories,

2 $${d}_{12}(t\gg {T}_{\lambda })\propto {\delta }^{\nu },\quad \quad {d}_{12}(t)=\langle |{{\bf{x}}}_{1}(t)-{{\bf{x}}}_{2}(t)|\rangle ,$$

may be used as a reliable indicator for chaos, where we denote with *d*_12_(*t* = 0) = *δ* the initial distance, and with *ν* the cross-distance scaling exponent.

For an illustration of how the long-term distance ${d}_{12}(t\gg {T}_{\lambda })$ depends on the initial distance *δ*, we show in Fig. 3(a) the time evolution of the distance *d*_12_ between pairs of trajectories, considering initial distances *δ* = 10^−8^ and *δ* = 10^−5^, as averaged over 10^4^ pairs. For strong chaos (red curves, *ρ* = *ρ*_1_) and PPC (green curves, *ρ* = *ρ*_2_) the long-term distance does not depend on the initial distance. The scaling exponent thus vanishes, *ν* = 0, for chaotic motion. The initial slope of the curves reflects the exponential divergence of chaotic trajectories within the time scale of the Lyapunov prediction time *T*_*λ*_. For the laminar flow (blue curves, *ρ* = *ρ*_3_) the long-term distance depends on the other side on *δ*, leading to a non-zero scaling exponent, *ν* ≠ 0.

For the results shown in Fig. 3(b) we have evaluated for every *ρ* considered the long-term distance *d*_12_(*t* = 200) starting from initial distances *δ* ∈ [10^−9^, 10^−4^], averaging each time over 10^3^ pairs of trajectories. We note that the scaling exponent *ν* can be extracted reliably from a linear regression of the data in a log-log plot when the initial distance *δ* is smaller than the distance of two neighboring attractors or parts of the same attractor. Additionally we note that the choice of *t* = 200 was selected such that *t* ≫ *T*_*λ*_ holds for the interval of *ρ* considered (cf. Table 1). Close to the period doubling transition to chaos, viz for $\rho \lesssim {\rho }_{{\rm{C}}}$, the maximal Lyapunov exponent becomes very small $({\lambda }_{{\rm{m}}}\lesssim {10}^{-2})$ and the Lyapunov prediction times large (*T*_*λ*_ > 200). In this case a larger time *t* ≫ *T*_*λ*_ would be needed.

The linear scaling *ν* = 1 observed for the limit cycle (*ρ* = *ρ*_3_) in Fig. 3(b) stems from the fact that any two orbits attracted by a limit cycle follow each other perpetually, with the average final separation being proportional to the initial separation. This relation can be motivated analytically using a local approximation to the attracting set in the normal form of limit cycles (cf. the Methods section).

For chaotic phases the long-term average distance settles on the other hand to a finite value determined by the extent of the attracting set, independently of the initial distance *δ*, leading to a vanishing scaling exponent *ν* = 0. As observed in Fig. 3(a) the time needed for strong chaos to reach long-term stationarity in *d*_12_(*t*) is proportional to the Lyapunov prediction time *T*_*λ*_. For PPC the long-term limit is however only reached for *t* > *T*_PPC_, where the decorrelation time *T*_PPC_, i. e. the time that a pair of trajectories needs to get fully uncorrelated, is significantly longer than both the quasi-period *τ* and the Lyapunov prediction time *T*_*λ*_. The scaling exponent *ν* ≈ 0 is hardly distinguishable from zero for measurements at time *t* ∈ [*T*_*λ*_, *T*_PPC_]. We remark that *d*_12_(*t* → ∞) is however determined by the overall extent of the attracting set in the limit of large times. For times *t* > *T*_*λ*_, right after the initial exponential decorrelation, the typical separation of two orbits *d*_12_(*t*) is of the order of the braid width (cf. insets in Fig. 2(b,c)).

In Fig. 1(c) the cross-distance scaling exponent *ν* for the entire range of *ρ* considered here is shown. We note, that the transition from chaos to laminar flow occurring at *ρ*_C_ ≈ 180.96 is accompanied by a sudden jump in *ν* from zero to one. This is quite remarkable, as the corresponding maximal Lyapunov exponent *λ*_m_, also shown in Fig. 1, becomes, on the other hand, continuously smaller when approaching *ρ*_C_ from the chaotic side.

The cross-distance scaling is a robust 0–1 test for chaos that also classifies PPC correctly^24^. For a further evaluation we applied it to the chaotic states found in previously studied neural networks^25^, which we generalized in size (with up to 300 dimensional phase spaces). We also examined the three-dimensional Shilnikov attractor^26^ (cf. Methods section), as it is similar to the Lorenz system, albeit with all degrees of freedom evolving on the same time scale. For both systems the test presented here worked without problems.

For a comparison we applied the Gottwald 0–1 test^27, 28^ to the three different dynamical regimes of the Lorenz system presented above (cf. Fig. 2). Using Gottwald’s method we were able to classify regular motion *ρ* = *ρ*_3_ and strong chaos *ρ* = *ρ*_1_ correctly, but not partially predictable chaos. For *ρ* = *ρ*_2_ even an exceedingly long run time, *t* = 10^6^, did not provide a clear result.

### Cross-correlation of initially close trajectories

An important point for real-world applications are the long-term repercussions of variations in the initial conditions. For concreteness we consider with

3 $${C}_{12}(t)=\langle ({{\bf{x}}}_{1}(t)-\mu )\cdot ({{\bf{x}}}_{2}(t)-\mu )\rangle /{s}^{2},$$

the cross-correlation function of two bounded and initially close-by trajectories **x**_1_(*t*) and **x**_2_(*t*). Here 〈…〉 denotes an average over initial conditions on the attractor sampled with the natural distribution, *μ* the center of gravity, and *s* the average extent of the attracting set,

4 $$\mu =\mathop{\mathrm{lim}}\limits_{T\to \infty }\frac{1}{T}{\int }_{T}^{2T}{\bf{x}}(t)\,{\rm{d}}t,\quad \quad {s}^{2}=\mathop{\mathrm{lim}}\limits_{T\to \infty }\frac{1}{T}{\int }_{T}^{2T}{[{\bf{x}}(t)-\mu ]}^{2}{\rm{d}}t\mathrm{.}$$

The cross-correlation is normalized to unity for close-by trajectories, i. e. for |**x**_1_(*t*) − **x**_2_(*t*)| → 0. For chaotic attracting sets the cross-correlation *C*_12_ vanishes in the long-term limit *t* → ∞, with a finite *C*_12_ ≠ 0 implying finite amounts of predictability.

For a geometric comparison we define the averaged square distance *D*_12_(*t*) = 〈[**x**_1_(*t*) − **x**_2_(*t*)]^2^〉 between two trajectories, which leads, when using (3), to

5 $${D}_{12}(t)=2{s}^{2}\mathrm{[1}-{C}_{12}(t\mathrm{)].}$$

For large cross-correlations *C*_12_ → 1 the two trajectories are close-by with respect to the overall extent *s* of the attracting region, in the sense that *D*_12_ ≪ *s* ^2^.

It is evident from Fig. 2, that the overall shape of the attractor changes little across the transition from laminar flow (*ρ* = *ρ*_3_) to chaos $(\rho ={\rho \,}_{2}^{\ast })$, and that the previously one dimensional attracting state (the limit cycles) does broaden to a closed chaotic braid. This behavior can also be viewed as chaotic wandering around limit cycles^1^.

**Figure 4:**
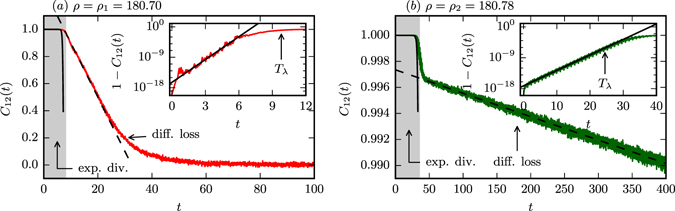
The cross-correlation *C*_12_ for pairs of trajectories with an initial distance *δ* = 10^−8^ and averaged over 10^4^ pairs over time. The data is for (**a**) strong chaos (*ρ* = *ρ*_1_ = 180.70) and (**b**) partially predictable chaos (*ρ* = *ρ*_2_ = 180.78); compare Fig. 1. For both cases we find an initial time interval of exponential decorrelation (shaded gray). The blow-ups magnifying that region show that the correlation *C*_12_ ≈ 1 initially decreases with $1-2.8\cdot {10}^{-13}{e}^{{\lambda }_{{\rm{C}}}t}$, *λ*_C_ = 5.17 (inset in (**a**)) and as $1-4.7\cdot {10}^{-14}{e}^{{\lambda }_{{\rm{C}}}t}$, *λ*_C_ = 1.04 (inset in (**b**)), as indicated by the respective solid lines. The linear decrease of *C*_12_ for intermediate times corresponding to a diffusive loss of predictability is ∝(−0.04*t*) in (**a**) and ∝(−1.8 · 10^−5^ *t*) in (**b**), as indicated by the respective dashed lines.

In Fig. 4 we present the time evolution of the cross-correlation *C*_12_ for the case of strong chaos, *ρ* = *ρ*_1_ in (*a*), and partially predictable chaos, *ρ* = *ρ*_2_ in (*b*). We note that *C*_12_ remains close to full predictability, ${C}_{12}\simeq 1$, within the respective time scales of the Lyapunov prediction time *T*_*λ*_. We have included in both panels of Fig. 4 fits to the cross-correlations of the form 1 − *c*exp(*λ*_C_ *t*), an approximation resulting from (5), where *λ*_C_ ≥ 2*λ*_m_^29^ (*c* is a fit parameter). *C*_12_ decreases linearly for times larger than *T*_*λ*_, saturating eventually to zero when full decorrelation is achieved^30, 31^:

6 $${D}_{12}(t)/2{s}^{2}=1-{C}_{12}(t)\propto \{\begin{array}{cc}{e}^{{\lambda }_{{\rm{C}}}t} & {\rm{f}}{\rm{o}}{\rm{r}}t < {T}_{\lambda }\\ t & {\rm{f}}{\rm{o}}{\rm{r}}t > {T}_{\lambda }\end{array}.$$

For strong chaos both the exponential divergence and the diffusive loss of predictability happen on the same time scale. We remark here that the evolution of the cross-correlation presented in Fig. 4 for the case of strong chaos bears a surprising similarity with the measured relative accuracy of weather forecasting over a period of two weeks^32, 33^.

For partially predictable attractors, *ρ* = *ρ*_2_, we find qualitatively the same behavior as for strong chaos, at least as matter of principle, with a dramatic separation of time scales setting however in beyond the initial phase of exponential divergence. The slope of the linear decrease is, as evident from Fig. 4(b), three orders of magnitude smaller in the partially predictable case (1.8 · 10^−5^ instead of 4 · 10^−2^).

PPC can be found also in systems controlled by a single microscopic time scale (cf. Methods section). We hence attribute the emergence of PPC to the fact, that the attractor is topologically equivalent, for *ρ* = *ρ*_2_, to elongated closed braids. Comparing the braid width from the insets in Fig. 2 to the linear distance *d*_12_(*t* = 200) in Fig. 3(b) (5 and 0.5 in comparison to 5.8 and 0.4 for *ρ* = *ρ*_2_ and $\rho ={\rho \,}_{2}^{\ast }$ respectively), we find that the initial exponential divergence occurs dominantly perpendicular to the braid. Once the separation of two trajectories has reached the braid width it can increase further only along the braid, which is in turn a diffusive process and hence slow. This means that the chaotic flow remains partially predictable for remarkable long times compared to the Lyapunov prediction time *T*_*λ*_. From the linear fit in Fig. 4(b) we estimate that it takes *T*_PPC_ ≈ 10^4^ until correlations vanish effectively for the partially predictable case *ρ* = *ρ*_2_.

The cross-correlation *C*_12_(*t* = 200) shown in the Fig. 1(d) vanishes for *ρ* < *ρ*_*p*_, which is hence a phase in which predictability is lost for times larger than the Lyapunov prediction time *T*_*λ*_. The ergodicity of pairs of trajectories is however broken for intermediate times *T*_*λ*_ < *t* < *T*_PPC_ in the PPC phase realized for *ρ*_*p*_ < *ρ* < *ρ*_C_. Partially predictable chaos is hence characterized both by positive Lyapunov exponents *λ*_m_ > 0 and by a finite predictability coefficient *C*_12_(*t* ≫ *T*_*λ*_) ≠ 0. This notion of predictability does naturally not exclude the possibility of finding additional finite time windows of predictability due to the presence of periods of quasi-laminar flow embedded in the overall chaotic time evolution^13^. Measuring the fractal dimension^3^ with the box-counting method we find that the attractors in the PPC phase have fractal dimensions slightly larger than two, as usual for the Lorenz system^34^.

The exceedingly slow loss of predictability occurring for *ρ* = *ρ*_2_ can be observed also in systems in which all defining dynamical parameters are of the same order of magnitude (cf. Methods section). The magnitude of the respective diffusion coefficient is hence only indirectly related, for the case of the Lorenz system, to the relative size of *β*, *σ* and *ρ* in (1). We also note that the neutral flow along the braids, i. e. the flow along the attractor which is characterized by a vanishing average ${\lambda }_{2}=\langle {\lambda }_{2}^{({\rm{l}})}\rangle =0$ of the second-largest local Lyapunov exponent ${\lambda }_{2}^{({\rm{l}})}$, is highly dispersive (cf. the Methods section). Additionally we remark that PPC manifests itself in a linear decrease of the amplitudes of the periodic oscillation of the auto-correlation function (cf. Methods section).

## Discussion

We have proposed here a new 0–1 test for the occurrence of chaos derived from the long-term scaling behavior of the distance between pairs of initially close trajectories. We find the 0–1 test to be extraordinarily robust and that chaotic dynamics may be partially predictable whenever two initially close trajectories remain within a finite but small distance for extended periods. Partial predictability occurs when the initial exponential divergence stops at length scales which are finite but substantially smaller than the overall extent of the attracting set. For the Lorenz system we found that residual predictability levels of the order of 99% are retained despite non-zero Lyapunov exponents^35^ and that the system is stable in this state against finite perturbations^7, 36^. The notion of partial predictability implies macroscopic predictability in terms of coarse grained predictions. Taking the case of weather forecasting, which is plagued notoriously by chaotic instabilities^13, 32, 37^, it may hence be possible to predict with confidence the formation of a low pressure area, to give an example, but not its exact extension and depth.

We have shown here that partial predictability is not a consequence of varying local Lyapunov exponents on the attracting set and that the averaged Lyapunov exponents in terms of the Lyapunov spectrum yield prediction times which are orders of magnitude smaller than the time scales observed for partial predictability. Partial predictability is essentially a consequence of topological constraints, e. g. when chaotic braids arise from a previous period doubling transition. PPC is hence expected to be found for a wide range of systems, such as enzyme reactions^38^ and models of asset pricing^39^. In this context we point out that indications for partially predictable chaos have been found recently in the phase space of the sensorimotor loop of simulated self-organized robots^40^. It would be interesting to investigate in further studies whether the concept of partial predictability, which does not require multiple time scales *per se*, could be generalized to time dependent snapshot^41, 42^ or pullback attractors^43^ arising in stochastic and/or driven chaotic systems.

The dynamical regimes discussed here – strong chaos, PPC and laminar flow – can be distinguished when combining the 0–1 test for chaos with an analysis of the long-term saturation plateau of the inter-trajectory cross-correlation function *C*_12_(*t*), which may be finite (for PPC and laminar flow) or zero (for strong chaos). We also stress that the three indicators – global maximal Lyapunov exponent, cross-correlation and cross-distance scaling – examined in this work rely on the evolution of initially close pairs of trajectories. These indicators can hence be evaluated by a straightforward manipulation of the data without the need to investigate further the nature of the attracting set. It is possible to automatize the computation of there examined 0–1 indicators – we provide a pseudo code routine in the Methods section – and to obtain thus a combined test capable to distinguish the three dynamical regimes characterizing dissipative autonomous dynamical systems.

## Methods

All computations that involved solving Eq. (1) were performed using a Runge-Kutta-Fehlberg algorithm^44^ of order 4/5 and step size *Δt* = 10^−3^. Testing the accuracy of the results by systematically varying *Δt* we found that the limitations due to the chaotic nature of the motion allow for reliable results for integration times up to *t* ~ 500.

### Derivation of the cross-distance scaling for limit cycles

Above we showed that the long-term distance ${d}_{\infty }=\mathop{\mathrm{lim}}\limits_{t\to \infty }{d}_{12}(t)$ for of two initially close-by trajectories scales linearly with the initial distance *δ* whenever the dynamics settles in an attracting limit cycle. For an analytic understanding of this observation we consider the two dimensional normal form for limit cycles in polar coordinates (*φ*, *r*)^6^,

7 $$\dot{\phi }={\rm{\Omega }}(r),\quad \quad \dot{r}=r({{\rm{\Gamma }}}^{2}-{r}^{2}),$$

where the time evolution of the angle *φ* is described by an arbitrary smooth function Ω(*r*) of the radius *r*, Expanding Eq. (7) to first order around the limit cycle *r*(*t*) ≡ Γ, viz using *r* = Γ + *ε*, we find

8 $$\dot{\phi }={\rm{\Omega }}({\rm{\Gamma }})+{\rm{\Omega }}^{\prime} ({\rm{\Gamma }})\varepsilon ,\quad \quad \dot{\varepsilon }=-2{{\rm{\Gamma }}}^{2}\varepsilon $$

for the behavior in the close neighborhood of the limit cycle, with Ω′ = dΩ/d*r* denoting the derivative with respect to the radius *r*. Substituting (*φ*, *ε*) → (*x*, *y*) and (Ω, Ω′, 2Γ^2^) → (*a*, *b*, *c*) we then obtain with

9 $$\dot{x}=a+by,\quad \quad \dot{y}=-cy$$

the Cartesian normal form of a limit cycle. The parameter *a* hence represents the base speed of the flow along the limit cycle, *b* the rate with which the flow parallel to the limit cycle changes with the distance *y* from the limit cycle, and *c* the time scale needed to relax to the attractor. The solution (*x*(*t*), *y*(*t*)) of the linearized system with the initial conditions (*x*, *y*)(*t* = 0) = (*x*_o_, *y*_o_) is given by

10 $$x(t)={x}_{{\rm{o}}}+at+\frac{b}{c}{y}_{{\rm{o}}}(1-{{\rm{e}}}^{-ct}),\quad \quad y(t)={y}_{{\rm{o}}}{{\rm{e}}}^{-ct}\mathrm{.}$$

As we are interested in the behavior of two initially close trajectories (of which both are close to the attractor), we consider two trajectories starting from (*x*_o_, *y*_o_) and (*x*_o_ + *δ*_*x*_, *y*_o_ + *δ*_*y*_). Here *δ*_*x*_ and *δ*_*y*_ denote the initial distances between the trajectories in their respective dimension and $\delta ={({\delta }_{x}^{2}+{\delta }_{y}^{2})}^{\mathrm{1/2}}$ the initial Euclidean distance between the trajectories. Both trajectories converge in the long-term limit *t* → ∞ to the limit cycle *y* → 0. The Euclidean distance between the trajectories is hence given by

11 $${d}_{12}(t)={({[{x}_{1}(t)-{x}_{2}(t)]}^{2}+{[{y}_{1}(t)-{y}_{2}(t)]}^{2})}^{\mathrm{1/2}}={({[{\delta }_{x}+\frac{b}{c}{\delta }_{y}\mathrm{(1}-{{\rm{e}}}^{-ct})]}^{2}+{\delta }_{y}^{2}{{\rm{e}}}^{-2ct})}^{\mathrm{1/2}}\mathrm{.}$$

The distance approaches a finite value in the long-term limit *t* → ∞, which we term the long-term distance

12 $${d}_{\infty }({\delta }_{x},{\delta }_{y})=\mathop{\mathrm{lim}}\limits_{t\to \infty }{d}_{12}(t)=|{\delta }_{x}+\frac{b}{c}{\delta }_{y}|,$$

where |·| denotes the modulus.

Averaging *d*_∞_(*δ*_*x*_, *δ*_*y*_) over a circle ${\mathscr{C}}$ centered around (*x*_o_, *y*_o_), defined by ${\delta }^{2}={\delta }_{x}^{2}+{\delta }_{y}^{2}$, we obtain

13 $$\langle {d}_{\infty }\rangle =\frac{1}{2\pi \delta }{\oint }_{{\mathscr{C}}}{\rm{d}}s\,{d}_{\infty }({\delta }_{x},{\delta }_{y})=\frac{2}{\pi }{(\frac{{b}^{2}}{{c}^{2}}+1)}^{\mathrm{1/2}}\delta \mathrm{.}$$

The average long-term distance 〈*d*_∞_〉 is hence proportional to the initial distance *δ*, with the constant of proportionality 2(*b* ^2^/*c* ^2^ + 1)^1/2^/*π* depending through *b*/*c* on the properties of the flow close to the limit cycle. The factor *b*/*c* can be smaller or larger than unity, implying that the long-term distance of the two trajectories may exceed the initial distance.

The normal form (9) describes the local flow close to a limit cycle. For the case of a non-uniform base velocity *a* = *a*(*x*) one need to generalize (13) by averaging over full periods.

### Choice of initial distances

**Figure 5:**
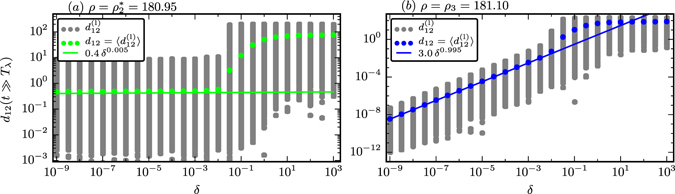
The scaling behavior of the averaged long-term distance *d*_12_(*t* = 200) (colored circles), see Eq. (2), with the respective fits on a log-log scale (fitted for *δ* < 10^−2^) (solid lines, cf. Fig. 3). The distribution of the non-averaged distances ${d}_{12}^{({\rm{l}})}(t=200)$, as obtained from 1000 initial conditions, are shown in addition (gray circles). (**a**) For PPC with $\rho ={\rho }_{2}^{\ast }=180.95$ one finds, in agreement with Fig. 3, a close to constant cross-distance scaling. (**b**) For a laminar flow with *ρ* = *ρ*_3_ = 181.10 the scaling exponent is *ν* ≈ 1 for distances *δ* < 10^−2^. In both cases the scaling breaks down when a symmetry related close-by attractor starts to attract a fraction of the orbits for *δ* > 10^−2^.

The cross-distance scaling (cf. Eq. (2)) is valid only when the two trajectories considered are attracted by the same attractor. This condition is satisfied for the values of *δ* considered in Figs 1 and 3, namely *δ* ∈ [10^−9^, 10^−4^], but not necessarily for larger values of *δ*, as illustrated in Fig. 5.

For the partially predictable chaotic attractor, with $\rho ={\rho \,}_{2}^{\ast }=180.95$ in Fig. 5(a), we find the expected scaling *ν* = 0.005 (solid line) for the average cross-distance *d*_12_(*t* = 200) (colored bullets) and initial distances up to *δ* ≈ 10^−2^. The gray dots represent the unaveraged cross-distances ${d}_{12}^{({\rm{l}})}(t=200)$, as obtained from distinct 1000 initial distances. For *δ* > 10^−2^ the two orbits start to end up in distinct attractors, as a second (symmetry related) PPC attractor exists close-by in phase space. After a crossover region *δ* ∈ [10^−2^, 1] one observes a second scaling plateau for *δ* > 1.

For the cross-distance scaling of the limit cycle, as presented for *ρ* = *ρ*_3_ = 181.10 in Fig. 5(b), we observe an equivalent behavior. In the limit of small initial distances *δ* < 10^−2^ we obtain in accordance with Fig. 3 the close to linear scaling *ν* = 0.995. Again there is a symmetry related limit-cycle attractor close-by in phase space, attracting a fraction of the orbits for *δ* > 10^−2^.

We note that attractors are surrounded, by definition, by a possibly small but in any case finite-sized basin of attraction^45^ and that the here proposed scaling analysis can be performed generically when considering initial conditions close enough to the attractor, separated by small initial distances *δ*. Basins of attraction may however fan out further away from the attracting set into complicated and possibly fractal structures^1, 46^.

### Global Lyapunov exponent

The results for the maximal average Lyapunov exponent *λ*_m_ presented above were computed from the averaged logarithmic distance 〈ln|**x**_1_(*t*) − **x**_2_(*t*)|〉 between two initially close-by trajectories over time. Alternatively one may evaluate *λ*_m_ using Benettin’s algorithm^21, 22^.

**Figure 6:**
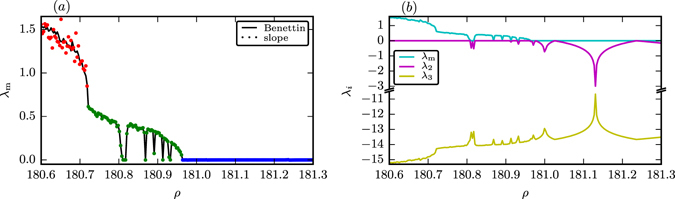
The Lyapunov exponents of the Lorenz system for *ρ* ∈ [180.6, 181.3]. (**a**) Comparing the maximal average Lyapunov exponent *λ*_m_ obtained using the method of Benettin^21^ (solid line) and from the initial slope of the averaged logarithmic distance 〈ln|**x**_1_(*t*) − **x**_2_(*t*)|〉 (dots). The distinct regimes are color coded (red/green/blue for strong chaos/PPC/laminar flow). (**b**) All three average Lyapunov exponents *λ*_*i*_ computed by the method of Benettin^21, 22^. (Note the break in the vertical scale).

In Fig. 6(a) we compare the average maximal Lyapunov exponent *λ*_m_ of the Lorenz system in the parameter range *ρ* ∈ [180.6, 181.3] as obtained by the linear slope of the averaged logarithmic distance between two initially close-by trajectories (colored dots) with the *λ*_m_ found when using Benettin’s method (solid line). For the latter method *λ*_m_ is given by the logarithmic ratio of an initial deviation from the attractor, here *δ* = 10^−8^, and its stretched time evolution. This quantity has been averaged equidistant in time for ~10^6^ points over the respective attractor with an integration time step of d*t* = 10^−3^. The data matches well for regular motion (blue) and for PPC (green). For strong chaos (red) there is however a non-negligible degree of scattering.

Using Benettin’s method we also computed the complete spectrum of Lyapunov exponents (*λ*_m_, *λ*_2_, *λ*_3_) for the Lorenz system in the considered parameter range, as depicted in Fig. 6(b). The largest and the second largest exponents are positive and zero for both chaotic regimes, as expected, and zero and negative respectively for a limit cycle. Summing up the exponents leads to ∑_*i*_ *λ*_*i*_ ≈ −13.67, which is in agreement with the phase space contraction rate −1 − *β* − *σ* = −13.66 of the Lorenz system.

### Distribution of local Lyapunov exponents

**Figure 7:**
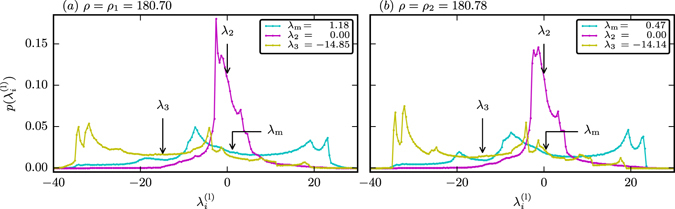
The probability distribution $p({\lambda }_{i}^{({\rm{l}})})$ of the local Lyapunov exponents ${\lambda }_{i}^{({\rm{l}})}$ for the Lorenz attractor; the global Lyapunov exponents *λ*_*i*_ given in the legend and indicated by arrows in the plot are the average of the respective distributions. The standard deviations are *s*_m_ = 14.3, *s*_2_ = 5.1 and *s*_3_ = 14.3 in (**a**) for *ρ* = *ρ*_1_ = 180.70 (strong chaos) and *s*_m_ = 14.6, *s*_2_ = 5.6 and *s*_3_ = 14.8 in (**b**) for *ρ* = *ρ*_2_ = 180.78 (PPC). A direct connection between the functional form of the distributions of Lyapunov exponents with the dynamics of PPC is not evident. For the PPC the variance of the local neutral exponent ${\lambda }_{2}^{(l)}$ is an order of magnitude larger (5.6 vs. 0.47) than the corresponding maximal Lyapunov exponent.

It is of interest to evaluate not only the averaged Lyapunov exponents, as presented in Fig. 6 and Table 1, but the full distribution of local Lyapunov exponents on the attracting set, both for the case of PPC and for strong chaos. For the data presented in Fig. 7 we computed the local Lyapunov exponents ${\lambda }_{i}^{({\rm{l}})}$ as the logarithm of the local expansion coefficient^22^ (the ratio of lengths of the orthogonalized deviation vectors after one simulation step and the initial deviation vectors), using Gram-Schmidt orthogonalization in every step. The local Lyapunov exponents were sampled equidistant in time with an integration time step of d*t* = 10^−3^, over a total time *T*_max_ = 10^5^, where the length of initial deviation vectors has been set to *δ* = 10^−8^ after every step of the simulation.

The distribution of local Lyapunov exponents covers a rather wide range of values as compared to the respective global Lyapunov exponents. There is hence no directly evident connection between the distribution of local Lyapunov exponents and the shape of the chaotic attractor, or to the characteristics of PPC. We emphasize that the local Lyapunov exponents presented in Fig. 7 are obtained from the stretching of the deviation vectors at every time step (not averaged) and that the global Lyapunov exponents ${\lambda }_{i}=\langle {\lambda }_{i}^{({\rm{l}})}\rangle$ correspond to the averages of the corresponding local exponents over the attracting set.

Figure 7 shows, most interestingly, that the neutral flow ${\lambda }_{2}=\langle {\lambda }_{2}^{(l)}\rangle =0$ is highly disperse around the chaotic braid. The speed of phase evolution covers several orders of magnitude. We have hence no evidence that the finding that *C*_12_ remains diffusive for prolonged time scales, as observed in Fig. 4(b), would result from an effective decoupling of a smooth phase and a chaotic radial evolution.

### Auto-correlations within PPC

**Figure 8:**
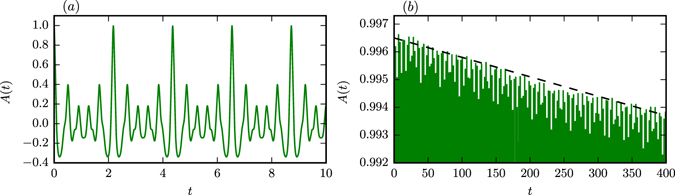
The autocorrelation *A*(*t*), as defined by Eq. (14), for *ρ* = *ρ*_2_ = 180.78 (PPC, compare Fig. 4(b)). (**a**) The oscillations resulting from looping around the braids are spaced by *τ* ≃ 2.2. (**b**) On longer timescales the auto-correlation decreases linearly, with the dashed line corresponding to 0.9965 − 7 · 10^−6^ *t*.

Instead of considering the properties of the cross-correlation between two trajectories one may study, alternatively, the autocorrelation function^8, 29, 47^

14 $$A(t)=\mathop{\mathrm{lim}}\limits_{T\to \infty }\frac{1}{T{s}^{2}}{\int }_{T}^{2T}{\rm{d}}t^{\prime} ({\bf{x}}(t^{\prime} )-\mu )({\bf{x}}(t^{\prime} -t)-\mu )$$

for the trajectory **x**(*t*) on the attractor, where *μ* and *s* denote, as defined by Eq. (4), respectively the mean and the average extent of the attracting set. In Fig. 8 we present *A*(*t*) for the PPC discussed in Fig. 4(b), i. e. for *ρ* = *ρ*_2_ = 180.78 (using *T* = 10^4^). One observes that the topology of the motion along fractally broadened braids shows up prominently in the autocorrelation function, with the quasi-period *τ* ≃ 2.2 of the attractor, determining the separation of the maxima of *A*(*t*).

The steady loss of predictability observed in Fig. 4(b) for PPC translates into a corresponding linear decrease (as indicated by the dashed line in Fig. 8(b)) of the heights of the local maxima of *A*(*t*). Using the autocorrelation function for the investigation of the slow decorrelation occurring in partially predictable chaos is hence possible, but plagued by the oscillatory nature of *A*(*t*). For this reason we concentrated in this study on the cross-correlation *C*_12_(*t*). The initial exponential decrease of correlations is furthermore only visible in the data for *C*_12_(*t*), but not for *A*(*t*) (compare Figs 4(b) and 8(b)).

### PPC in a system without separation of scales

**Figure 9:**
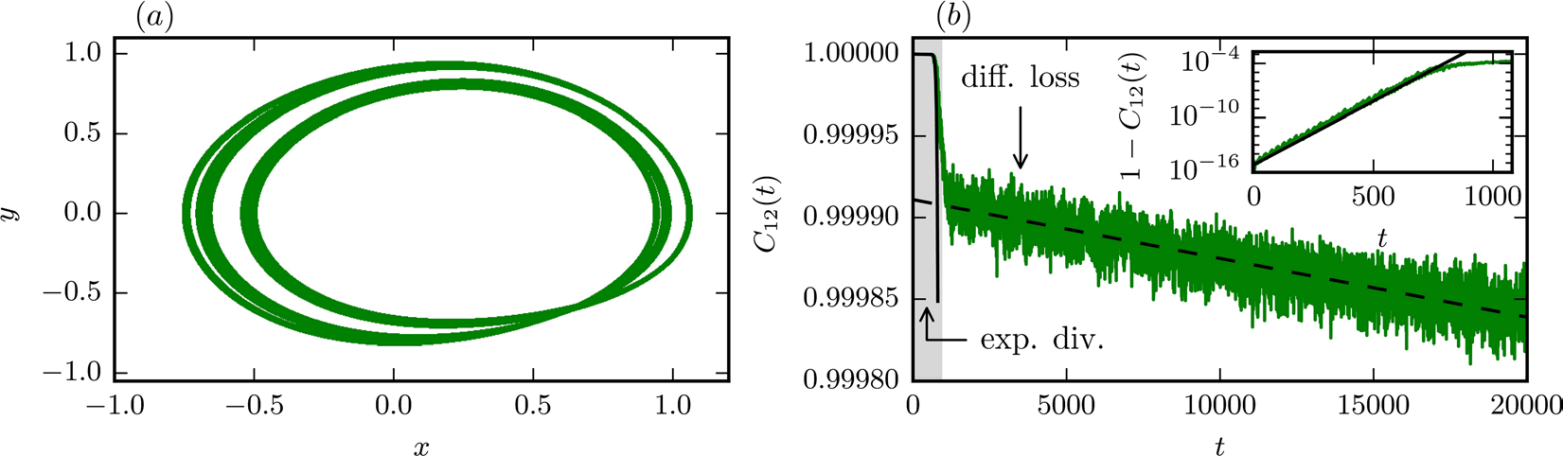
The attractor of the dynamical system described by Eq. (15), showing partially predictable chaos for *b* = 0.3783. (**a**) The projection to the *x* − *y* plane showing chaotic braids. (**b**) The cross-correlation *C*_12_, which decreases first exponentially (gray shaded region), as indicated by the fit $1.3\cdot {10}^{-9}{{\rm{e}}}^{{\lambda }_{{\rm{C}}}t}$, *λ*_C_ = 0.032, (solid line). For larger times the cross-correlation decreases linearly with a slope ∝3.6 · 10^−9^ *t* (dashed line).

Our choice of the parameters *β* = 8/3, *σ* = 10 and *ρ* > 180 for the Lorenz system (1) resulted in parameters and hence possibly also in time scales of distinct orders of magnitude. The question then arises if the observed large timescale for the final decorrelation process in the partially predictable phase may be a consequence of occurrence of distinct microscopic time scales. In order to rule out this scenario we have investigated with^26^

15 $$\dot{x}=y\,,\quad \quad \dot{y}=z,\quad \quad \dot{z}={x}^{3}-x-y-bz$$

a system for which the defining parameters are with *b* ∈ [0, 1] all of the same order of magnitude. For *b* = 0.3783 we find the attractor shown in Fig. 9(a), which classifies as PPC (partially predictable chaos). The average maximal Lyapunov exponent is positive (*λ*_m_ = 0.01, *λ*_2_ = 0, *λ*_3_ = −0.39), with the chaotic motion being restricted to braids of finite width. For slightly larger *b* > 0.3783 a cascade of period-doubling (halving) bifurcations occurs^26^.

The cross-correlation shown in Fig. 9(b) decays extraordinarily slow (cf. Fig. 4(b)), with a slope of the order of 10^−9^, implying that the retention of partial predictability cannot be attributed to the occurrence of large intrinsic time scales. The usual initial drop of the cross-correlation by about 0.01% within the Lyapunov prediction time *T* ≃ 805 is however present.

### Automation of the testing procedure

As the cross-distance scaling exponent and the finite time cross-correlation can be used as 0–1 tests for chaos and PPC respectively, it is possible to determine whether a system is chaotic, partially predictable or regular, by an automatized procedure. From the time evolution of pairs of trajectories one then determines the maximal Lyapunov exponent *λ*_m_, the cross-distance scaling exponent *ν* and the finite time cross-correlation *C*_12_(*t* ≫ *T*_*λ*_), where the latter two will be practically binary. The three different dynamics can be characterized subsequently by the criteria listed in Table 2.

1. First one needs to localize the attractor and estimate an upper bound for the initial distance *δ* ≪ 1 (cf. the Methods section).
2. One then computes the average maximal Lyapunov exponent *λ*_m_ from the slope of the averaged logarithmic distance $\langle \mathrm{log}|{{\bf{x}}}_{1}(t)-{{\bf{x}}}_{2}(t)|\rangle$, where the average is performed over pairs of trajectories **x**_1_(*t*) and **x**_2_(*t*) starting from a fixed initial distance *δ* ≪ 1. Other methods, like Benettin’s algorithm^21^, may be used alternatively.
3. The Lyapunov prediction time *T*_*λ*_ is then given by the inverse of the Lyapunov exponent.
4. Next, one computes the average Euclidean distance *d*_12_(*t* = 10*T*_*λ*_) for a range of initial distances *δ* ≪ 1, from which the scaling exponent *ν* is extracted using *d*_12_(*t* > *T*_*λ*_) ~ *δ* ^*ν*^. The flow is chaotic for *ν* = 0 and regular for *ν* = 1.
5. For the case of chaotic flows one measures additionally the finite time cross-correlation *C*_12_(*t* = 10*T*_*λ*_) for pairs of trajectories with initial distance *δ* ≪ 1. The finite time cross-correlation *C*_12_(*t* = 10*T*_*λ*_) is close to unity and zero respectively for partially predictable and for strong chaos.

**Table 2:** Combining the scaling exponent *ν* and the level of the cross-correlation at finite time, *C*_12_(*t*), allows to classify the three possible types of dynamics (compare Fig. 1).

*ν*	*C*_12_(*t*)	dynamics
0	0	strong chaos
0	1	PPC
1	1	laminar flow
